# Supplementary material for: Splice-Junction-Based Mapping of Alternative Isoforms in the Human Proteome
Source: Cell Rep. Author manuscript; Available in PMC 2020 Jan 15. (PMC6961840; doi:10.1016/j.celrep.2019.11.026)
Supplement: 2 [file NIHMS1546469-supplement-2.zip › DF1/PXD010154/Heart/Heart_88_AFMID_QQTLCQGEWK.pdf]

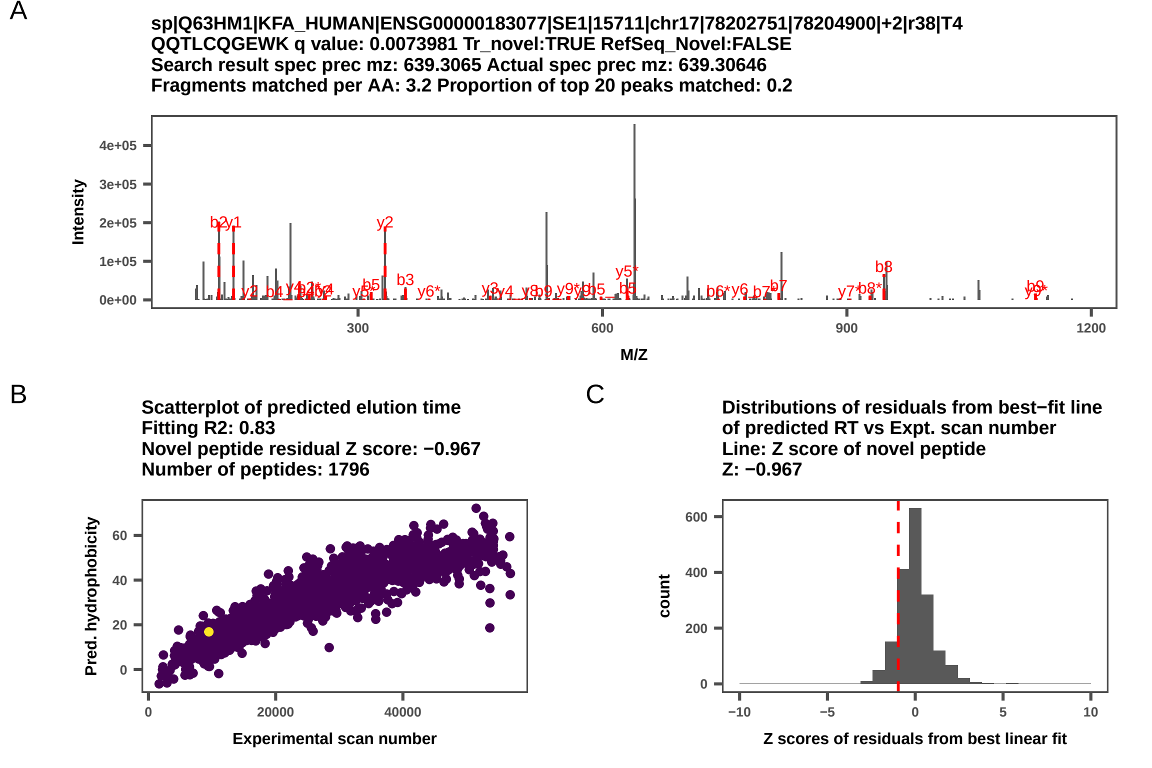
A
sp|Q63HM1|KFA_HUMAN|ENSG00000183077|SE1|15711|chr17|78202751|78204900|+2|r38|T4
QQTLCQGEWK q value: 0.0073981 Tr_novel:TRUE RefSeq_Novel:FALSE
Search result spec prec mz: 639.3065 Actual spec prec mz: 639.30646
Fragments matched per AA: 3.2 Proportion of top 20 peaks matched: 0.2
Intensity
4e+05
3e+05
2e+05
1e+05
0e+00
b2
y1
y2
b3
y5*
b8
300
600
900
1200
M/Z
B
Scatterplot of predicted elution time
Fitting R2: 0.83
Novel peptide residual Z score: −0.967
Number of peptides: 1796
Pred. hydrophobicity
60
40
20
0
20000
40000
Experimental scan number
C
Distributions of residuals from best−fit line
of predicted RT vs Expt. scan number
Line: Z score of novel peptide
Z: −0.967
count
600
400
200
0
−10
−5
0
5
10
Z scores of residuals from best linear fit
